# Single-Cell Genetic Analysis Using Automated Microfluidics to Resolve Somatic Mosaicism

**DOI:** 10.1371/journal.pone.0135007

**Published:** 2015-08-24

**Authors:** Keith E. Szulwach, Peilin Chen, Xiaohui Wang, Jing Wang, Lesley S. Weaver, Michael L. Gonzales, Gang Sun, Marc A. Unger, Ramesh Ramakrishnan

**Affiliations:** Fluidigm Corporation, South San Francisco, California, United States of America; University of Bonn, Institut of experimental hematology and transfusion medicine, GERMANY

## Abstract

Somatic mosaicism occurs throughout normal development and contributes to numerous disease etiologies, including tumorigenesis and neurological disorders. Intratumor genetic heterogeneity is inherent to many cancers, creating challenges for effective treatments. Unfortunately, analysis of bulk DNA masks subclonal phylogenetic architectures created by the acquisition and distribution of somatic mutations amongst cells. As a result, single-cell genetic analysis is becoming recognized as vital for accurately characterizing cancers. Despite this, methods for single-cell genetics are lacking. Here we present an automated microfluidic workflow enabling efficient cell capture, lysis, and whole genome amplification (WGA). We find that ~90% of the genome is accessible in single cells with improved uniformity relative to current single-cell WGA methods. Allelic dropout (ADO) rates were limited to 13.75% and variant false discovery rates (SNV FDR) were 4.11x10^-6^, on average. Application to ER-/PR-/HER2+ breast cancer cells and matched normal controls identified novel mutations that arose in a subpopulation of cells and effectively resolved the segregation of known cancer-related mutations with single-cell resolution. Finally, we demonstrate effective cell classification using mutation profiles with 10X average exome coverage depth per cell. Our data demonstrate an efficient automated microfluidic platform for single-cell WGA that enables the resolution of somatic mutation patterns in single cells.

## Introduction

Genetic mosaicism in somatic cells occurs naturally in an array of normal biological processes and contributes significantly to disease etiologies, particularly tumorigenesis [1–13]. Cancers are known to manifest as dynamic evolutionary processes in which intratumor genetic and phenotypic diversity is an inherent feature of the disease [3]. Genetic diversity amongst cells of a tumor can subsequently lead to clonal selection and is a known source of therapeutic escape, creating challenges for personalized monitoring and treatment. While large-scale projects have performed extensive analysis of somatic mutations across cancer types [14, 15], such studies lack the ability to define how the identified mutations segregate amongst individual cells. As a result, identification of somatic mutations from bulk DNA precludes the ability to determine how such mutations may interact to produce distinct phenotypes, fundamentally masking the features presented by the subclonal phylogenetic architecture. For these reasons, genetic analysis at single-cell resolution is becoming increasingly recognized as an important means by which to accurately characterize cancers. Despite these emerging themes, comprehensive methods for accurate single-cell genetics have been lacking.

In order to achieve accurate genetic analysis in individual cells, genomic DNA must be amplified with breadth and precision such that various modes of mutation can be captured (i.e., single-nucleotide variants (SNVs), insertions/deletions (INDELS), copy number variations (CNVs), and structural variants (SVs)). Currently, single-cell genetic analyses have generally been implemented using either PCR-based or isothermal multiple displacement amplification (MDA). PCR-based methods have better amplification uniformity at the expense of genomic coverage for CNV detection and result in ~10-fold more single-nucleotide errors than MDA-based methods. As an example, degenerate oligonucleotide-primed PCR (DOP-PCR) allows for detection of CNVs, but only achieves ~10% coverage genome-wide [7], leading to a lower detection rate of SNVs and SVs. Improved temperature cycling methods, including multiple annealing and loop-based amplification cycling (MALBAC)[12, 13], offer broader genomic coverage while maintaining uniformity sufficient for CNV analysis but can still result in ≥ 30% base dropout [11–13], again sacrificing sensitivity in detecting single-nucleotide mutations. On the other hand, isothermal MDA using high-fidelity Φ29 DNA polymerase allows for rapid and broad amplification across the genome with fidelity that is an order of magnitude higher than PCR-based approaches, making it particularly well-suited for identification of point mutations. However, if not controlled properly, MDA is known to result in amplification biases and nonuniformity that can prevent accurate genotyping. Recent advancements in MDA-based whole genome amplification (WGA) from single cells have included using G2/M cells to increase input DNA[11]; limiting reaction volumes to the nanoliter scale to improve primer binding kinetics during the early phases of amplification [16]; limiting amplification time in order to minimize biases [11, 16]; or treating the DNA post-amplification to facilitate downstream library generation [16] as a means to ultimately improve coverage uniformity across the genome while maintaining high fidelity.

Here we present an automated workflow for the capture, lysis, and MDA-based WGA of genomic DNA from up to 96 single cells at a time using nanoliter-scale reactions within integrated fluidic circuits (IFCs) that are fabricated by multilayer soft lithography [17] and are controlled by an automated microfluidic instrument. This workflow produces ~150–250 ng of DNA per cell in about 8 hours, enabling multiple downstream applications from the same cell, including targeted resequencing, whole exome sequencing (WES), and/or whole genome sequencing (WGS). We first established this approach using karyotypically normal cells, demonstrating high cell capture rates, low sample cross-contamination, and uniform DNA yields from captured single cells. We then applied our approach to individual cells from the ER-/PR-/HER2+ breast cancer cell line CRL2338/HCC1954 as well as matched normal B-lymphoblasts (CRL2339/HCC1954BL) isolated from the same individual. Single-cell WGS on six cells followed by WES on 100 matched normal/tumor cells in order to assess amplification uniformity, ADO, and rates of single-nucleotide errors compared to current single-cell WGA methods. Our method represents improvements on breadth of genomic coverage and uniformity of genomic coverage over previously reported methods, enabling accurate genotyping at single-cell resolution on an automated platform.

## Materials and Methods

### Cell culture conditions

GM12752 cells were obtained directly from Coriell and cultured in RPMI-1640 (ATCC) supplemented with 2mM L-glutamine and 15% FBS. CRL-2338 (HCC1954, ductal carcinoma) and CRL-2339 (HCC1954-BL, Normal B-lymphoblasts, immortalized) cells were obtained directly from ATCC and cultured in RPMI-1640 (ATCC) supplemented with 10% Fetal Bovine Serum. Cells were subcultured at a ratio of 1:4 to 1:8 2–3 times per week using 0.25% Trypsin (0.53M EDTA).

### Single-cell capture and validation of cell viability

Single cells were prepared and captured according to the Fluidigm protocol (Using C1 to generate libraries for DNA sequencing, PN 100–7135). GM12752 and CRL2339 cells were used on C1 IFCs with 10-17um capture sites. CRL2338 cells were captured on one 10-17um C1 IFC and one 17-25um C1 IFC. Captured cells were stained using the LIVE/DEAD Cell Viability/Cytotoxicity Assay Kit for mammalian cells (Life Technologies, L-3224) and imaged on a LeicaDMI 4000B microscope in the bright field, GFP, and Cy3 channels using Surveyor V7.0.0.9 MT software (Objective Imaging) to verify the presence and viability of single live cells in isolated chambers.

### Whole genome amplification

Whole genome amplification was performed at 38°C for 2hrs according to the Fluidigm protocol (Using C1 to generate libraries for DNA sequencing, PN 100–7135). Briefly, cells were captured in the C1 IFC (4.5nL) and lysed with 9nl of 0.4M potassium hydroxide followed by neutralization with 18nl of 0.2M hydrochloric acid. The neutralized mixture was then combined with 270nl of MDA reaction mix and incubated at 38°C for 2hrs with active mixing performed on C1 System. WGA DNA yields were quantified using the Quant-iT PicoGreen dsDNA Assay Kit (Invitrogen, P7589). WGA concordance with the presence of a live cell was determined using a threshold of 108ng from single live cells, verified from images of the captured cells subjected to the LIVE/DEAD cell viability assay. The threshold of 108ng was established by assessing WGA yields visually confirmed empty capture sites and determining the minimum WGA yield that. Whole genome amplified DNA was used in a modified Tn5 transposon-based library preparation using the Illumina Nextera DNA Sample Preparation Kit as described in the Fluidigm protocol (Using C1 to generate libraries for DNA sequencing, PN 100–7135). A modified protocol for whole exome enrichment was performed using the Illumina Nextera Rapid Exome Capture Kit (Standard 37MB, FC-140-1003) as described in Fluidigm protocol (Using C1 to generate libraries for DNA sequencing, PN 100–7135).

### Sequencing of whole genome and whole exome libraries

Whole genome and whole exome libraries were sequenced on an Illumina HiSeq 2000 instrument using 100bp paired-end reads (2X 100bp). Total read yields and sequence yields are listed in S2 Table along with the appropriate NCBI GEO and SRA accessions. All sequencing data associated with this manuscript has been deposited to the NCBI Sequence Read Archive under BioProject accession PRJNA287813.

### Alignment and processing of sequence data

Sequencing reads were aligned to GRC37/hg19 reference using bowtie2 (version 2.0.0-beta7) [18, 19] followed by local realignment and base-quality recalibration with GATKv2.6 [20]. Duplicates were removed using Picard *MarkDuplicates* (http://picard.sourceforge.net, picard-1.92) and only unique reads were retained for downstream analyses.

### Breadth of genomic coverage analysis

Uniquely aligned reads were randomly sampled using Picard *DownSampleSam* and depth of coverage at each base in the reference genome was determined using bedtools coverage–d (bedtools v2.17.0) [21].

### Read distribution uniformity analysis

Uniquely aligned reads were assigned to 100kb bins throughout the genome using bedtools coverage–count (bedtools v2.17.0). Lorenz curves were generated by calculating the cumulative fraction of reads as a function of the cumulative fraction of the genome. To quantify deviation from ideal uniformity, Gini coefficients were calculated as the ratio of the area under the curve for a given sample to the area under the ideal diagonal.

### Single-cell SNV identification

SNV calls were made using GATKv2.6 *UnifiedGenotyper*. A multi-sample call with default settings was made using all single cells and bulk genomic DNA controls at once in order to capture sites genotyped in at least one sample across all samples tested. This ensures sequence information for reference calls and non-covered sites was captured across the entire population of cells tested.

### Calculation of the variant false discovery rate

To estimate the rate of false positive variant identification, we identified high-confidence homozygous reference sites concordant in bulk genomic DNA controls. High-confidence sites were defined as sites with a sequencing depth of coverage ≥ 20 at which there was no evidence of a non-reference allele in order to minimize the chance of missing a low-frequency variant allele. In total, we identified 5,487,871 of these sites. We then measured the frequency of non-reference alleles detected at these sites in single-cell whole-genome-amplified DNA to determine an upper bound of the variant false discovery rate.

### Calculation of allelic dropout rates

Allelic dropout is defined as the rate at which one allele or the other is lost at high-confidence heterozygous sites identified in bulk genomic DNA. High-confidence heterozygous sites were defined as those concordant amongst bulk genomic DNA controls with a sequencing depth ≥ 10. In total, 7,414 of these sites were identified and used to determine allelic dropout. ADO calculated in this way serves as an upper bound on the true allelic dropout rate, since normal heterogeneity will contribute to the ADO rate.

### Identification and characterization of mutations in CRL2338/HCC1954 cells

Mutations were identified by requiring a sequencing depth ≥10 and the variant to be present ≥3 single cells, corresponding to a probability of 2.59x10^-4^ false variants per single-cell WES, based on the SNV FDR. Additionally, a Fisher’s exact test was applied to the number of variant events in the CRL2338/HCC1954 and CRL2339/HCC1954BL cells, requiring each variant to have p-value ≤ 0.05 (one-sided test, requiring the alternative hypothesis to be less). The variant allele frequency in CRL2338/HCC1954 cells was required to be ≥15%, and the variant allele frequency in CRL2339/HCC1954BL normal bulk genomic DNA controls was required to be ≤5%. Clustered mutations ≤10bp of one another were removed to avoid false positive caused by misalignment. Mutations were then annotated against the canSAR database and the cancer5000 dataset to identify those within genes previously found to have mutations in HCC1954 cells or various types of primary tumors, respectively. Hierarchical clustering based on Hamming distance was used for cell classification. Dendrograms and heatmap representations of the genotypes were generated using an open source R package developed in conjunction with this work (Singular Analysis Toolset, freely available at https://www.fluidigm.com/software).

### Analysis of mutations in down-sampled single-cell WES

Aligned reads were randomly down-sampled from the full dataset using Picard *MarkDuplicates* and genotypes at the mutations identified were called using GATK *UnifiedGenotyper* without filtering the genotype calls. The percentage of mutations genotyped was determined as the percent of sites genotyped in a given down-sampled dataset compared to the full dataset (~27X average depth of coverage). Mutation concordance was quantified as the percentage of sites correctly called mutant (HET or HOM ALT) in a given down-sampled dataset compared to the full dataset (~27X average depth of coverage), requiring a HET or HOM ALT call at the same site.

### Correlation between bulk genomic DNA and single-cell ensemble VAFs

To assess the correlation in VAFs between bulk genomic DNA controls and the single-cell ensemble data, we first identified all variants that were concordant in all bulk genomic DNA controls, requiring a sequencing depth ≥20. We then calculated a weighted average for the VAFs at these same positions in the single-cell data for variants with sequencing depth ≥10 as the ensemble.

VAF correlations for the down-sampling experiments were performed using unfiltered genotype calls from the CRL2338/HCC1954 WES data for each mutation in each cell (for both the set of 323 mutations and the set of 84 validated mutations).

### Data handling and statistics

To determine the number of cells in which a mutation was required to be present for that mutation to be considered true, we applied the variant false discovery rate to a binomial test, calculated as:

$$P\left(i\right)=C_{n}^{i}p^{i}{\left(1-p\right)}^{\left(n-i\right)}$$

Where the probability *P(i)* is the probability under the binomial distribution, *p* is the SNV FDR (4.11x10^-6^), *n* is the number of cells tested (n = 50), and *i* is the number of cells with a mutation (ranging from 1–10) (S3 Table). We chose a minimum threshold of 3 cells, which corresponded to a *P(i)* = 1.09x10^-11^. Additionally, we calculated the expected number of mutations detected by chance given the exome size. If a variant is observed in at least three cells, less than one false variant per single cell (2.59x10^-4^) is expected. A one-sided Fisher’s exact test was then applied to ensure the mutation was more frequently found in the CRL2338/HCC1954 tumor cells than in the normal CRL2339/HCC1954BL cells (one-sided test, requiring the alternative hypothesis to be less, p-value ≤ 0.05). The variant allele frequency in CRL2338/HCC1954 cells was required to be ≥15% and the variant allele frequency in CRL2339/HCC1954BL normal bulk genomic DNA controls was required to be ≤5%.

Cell classification was performed using hierarchical clustering of genotypes based on Hamming distance. All analyses were done using an open source R package developed in conjunction with this work, the Singular Analysis Toolset. This package is freely available at https://www.fluidigm.com/software.

For the single-cell WGS studies, no statistical methods were used to determine sample sizes, but our sample sizes exceed those currently reported in the literature for single-cell WGS, allowing for comparative analyses of the results. Similarly, no statistical methods were used to determine sample sizes for single-cell WES studies, although these numbers are also consistent with those previously reported in the literature. One sample from the set of 50 CRL2339/HCC1954 cells was excluded from the analysis as a result of low read yield (<4 million reads).

## Results

**Fig 1:**
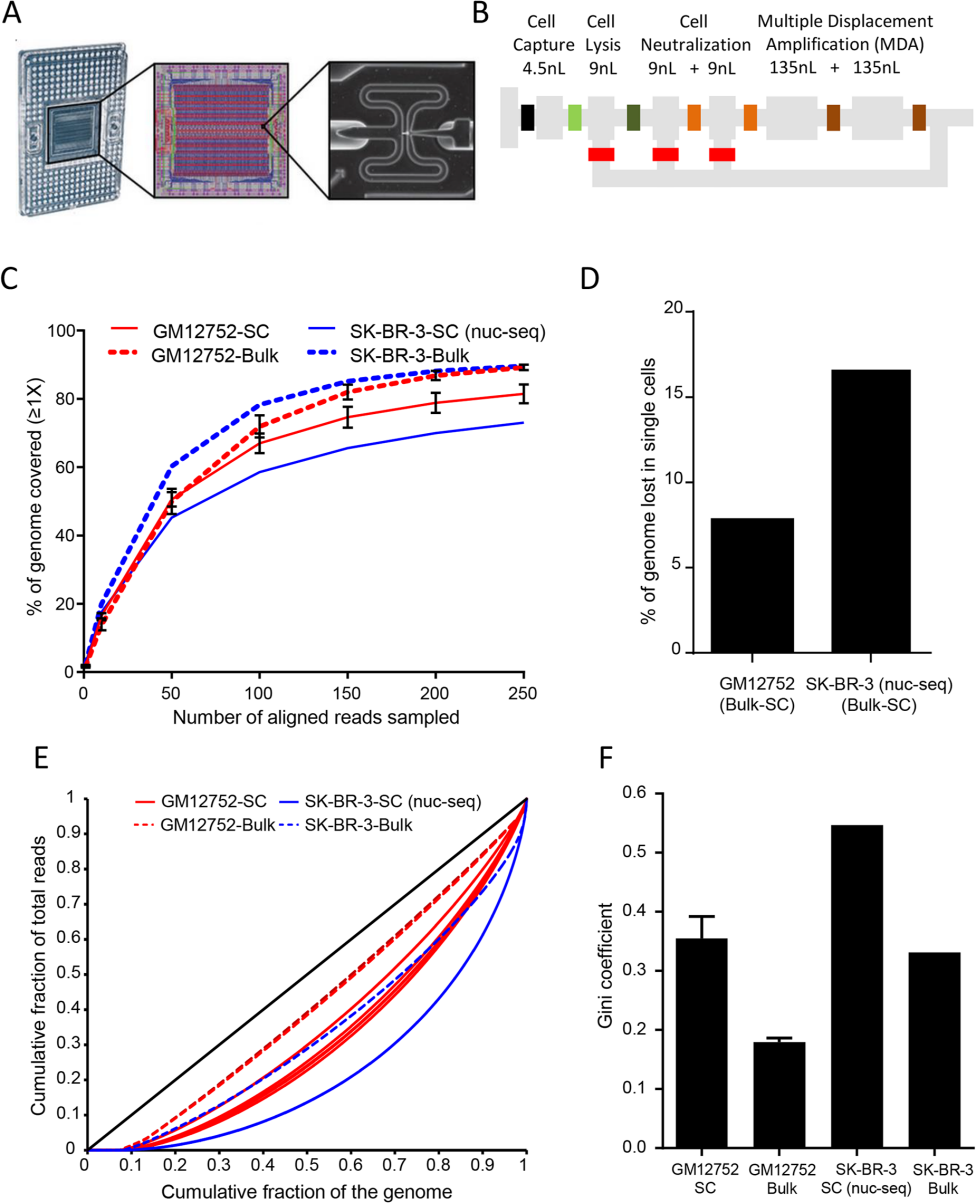
C1 Single-cell whole genome amplification workflow and performance on normal B-lymphoblasts. **(A)** Illustration of the C1 Integrated Fluidic Circuit (IFC) for single-cell capture and WGA. Left, IFC with carrier; reagents, and cells are loaded into dedicated wells, and the 96 WGA products are exported to other dedicated carrier wells. Middle, diagram of the IFC; connections between the polydimethylsiloxane microfluidic chip and carrier (pink circles), control lines (red), fluidic lines for WGA chemistry (blue), and bus lines connecting the control lines (green) are shown. Right, example of a single cell captured in one of the 96 4.5nL capture sites on the IFC. (**B)** Schematic of a C1 IFC reaction site. The reaction line is colored in gray with isolation valves shown in varied colors. Reagents are delivered through a common central bus line (shown on the far left). Each reaction begins in the 4.5-nl capture site. Alkaline lysis reagent expands the reaction to the first 9-nl chamber (13.5nL total), allowing for release and denaturation of genomic DNA. Neutralization of the lysis buffer occurs in the second and third 9-nl chambers (31.5nL total). Finally, the two 135-nl reaction chambers are used for WGA (301.5nL total). After the addition of WGA reagents, the contents of the reaction line are pumped in a loop using a bypass line (bottom) for mixing. After the WGA reaction is complete, each single-cell reaction product exits the chip using a dedicated fluidic path to the carrier (shown on the right). (**C)** Read-depth-dependent breadth of genomic coverage (≥1x) for C1 single-cell WGA and nuc-seq, as well as the corresponding bulk genomic DNA controls. Reads were sampled at various increments, up to 250 million (at which point coverage gains were minimal with additional sequence), and the fraction of the genome covered at ≥1x was determined to quantify the portion of the genome accessible with an equivalent number of reads across all samples. GM12752 single cells, n = 5, mean ± SD. GM12752 bulk genomic DNA, n = 2, mean ± SD. (**D)** C1 single-cell WGS on normal B-lymphoblasts (GM12752) resulted in a 6.95% loss in coverage compared to the bulk genomic DNA control, while nuc-seq (MDA on ~4N G2/M nuclei) resulted in 16.6% less coverage in single cells compared to a bulk genomic DNA control. (**E)** Read distribution uniformity represented as Lorenz curves for individual GM12752 cells (solid red, n = 5) and a SK-BR-3 single cell (SK1, solid blue), along with corresponding bulk genomic DNA controls (GM12752-gDNA, dotted red, and SKP, dotted blue). The ideal black diagonal line represents perfect uniformity. (**F)** Gini coefficients (*G*) derived from the Lorenz curves to quantify the dispersion of reads throughout the genome for GM12752 single cells (n = 5), GM12752 bulk genomic DNA (n = 2), SK-BR-3 single cell (SK1, n = 1), and SK-BR-3 bulk genomic DNA control (SK-P, n = 1). *G* = 0 is perfect uniformity, *G* = 1 is complete non-uniformity.

We implemented single-cell WGA using Φ29-mediated MDA on the Fluidigm C1 system, an automated hands-free microfluidics platform that uses IFCs for the capture, lysis, and WGA of DNA from single-cells within sequestered nanoliter scale reaction chambers (Fig 1A and 1B). Efficient single-cell genetic analysis requires high cell capture rates coupled with minimal contamination of nonspecific DNA. Minimizing the amount of sample contamination, in particular, is an important consideration due to the low amounts of starting material and the sensitivity of the amplification methods to exogenous DNA [22]. By sequestering captured single cells in valve-controlled microfluidic architecture, such cross-contamination can be reduced, limiting sample losses. Further, live/dead cell staining of sequestered cells can be used to verify the presence of single viable cells for downstream analyses. To test cell capture efficiency and subsequent WGA on the C1 system, we first used cultured B-lymphoblasts from a karyotypically normal male individual (GM12752, Coriell) to minimize potential variability in WGA due to genomic instability amongst individual cells. We observed cell capture rates of 80±6 cells per 96 IFC capture sites (S1 Table, 82.7%, n = 1,248 capture sites on 13 C1 IFCs, mean±SD). Additionally, we find that contamination is, in fact, low within the C1 IFC, as evidenced by a 97.3% concordance between WGA DNA yield and the presence/absence of a live cell visually confirmed using a viability assay (see S1 Table and materials and methods). Total WGA DNA yields for single live cells were 209 ± 69 ng (mean ± SD) (S1 Table) and WGA amplicon length is normally distributed around a peak of ~10 kb, ranging from 1.25–25 kb (S1A and S1B Fig), consistent with previous reports for Φ29 mediated MDA of human DNA [22, 23]. This corresponds to an amplification gain of 3.2 x 10^4^ (S1 Table), which is within the range where amplification uniformity is similar between nanoliter-scale MDA and PCR-based methods [22, 23]. Together, these results demonstrate efficient single-cell capture and amplification of genomic DNA with minimal sample cross-contamination using the C1 system.

To determine the breadth and evenness of genomic coverage using our single-cell WGA, we generated libraries from a subset of cells using Tn5 transposase-based-adapter tagging and performed WGS. Libraries were generated from five single cells and two non-amplified bulk genomic DNA controls (GM12752, Coriell). We quantified the breadth of genomic coverage by sub-sampling an equal number of reads from each sample and determining the fraction of the genome covered at ≥1X at various levels of sequencing depth (Fig 1C). Coverage was also directly compared to previously published data generated from SK-BR-3 breast cancer cells using an MDA based approach that takes advantage of using flow sorted G2/M nuclei (nuc-seq, ~4N genomic content) to increase the number of genomic copies and amount of DNA input to WGA [11]. Using a total of 250 million mapped reads, we observed 7.8% ± 2.8% (n = 5, mean ± SD) loss of coverage in C1 single-cell WGS compared to the bulk genomic DNA control. In comparison, an equivalent number of reads derived from nuc-seq had a 16.6% loss relative to a bulk genomic DNA control with coverage equivalent to the GM12752 bulk DNA controls (Fig 1D, SK-BR-3 population control 89.6%, GM12752 population control 89.3%±1.4%, n = 2, mean ± SD). Amplification uniformity across the genome was quantified by calculating Lorenz curves and deriving Gini coefficients (*G*), where *G* = 0 indicates perfect uniformity and *G* = 1 indicates complete lack of uniformity. WGA using the C1 system yielded *G* = 0.36±0.04 (mean ± SD), while *G* = 0.55 (mean ± SD) for nuc-seq. These data demonstrated that compared to nuc-seq, even with half the amount of starting material, our WGA results on the C1 system offer significantly improved uniformity across a larger fraction of the genome without requiring presorting of G2/M nuclei (~4N cells).

**Fig 2:**
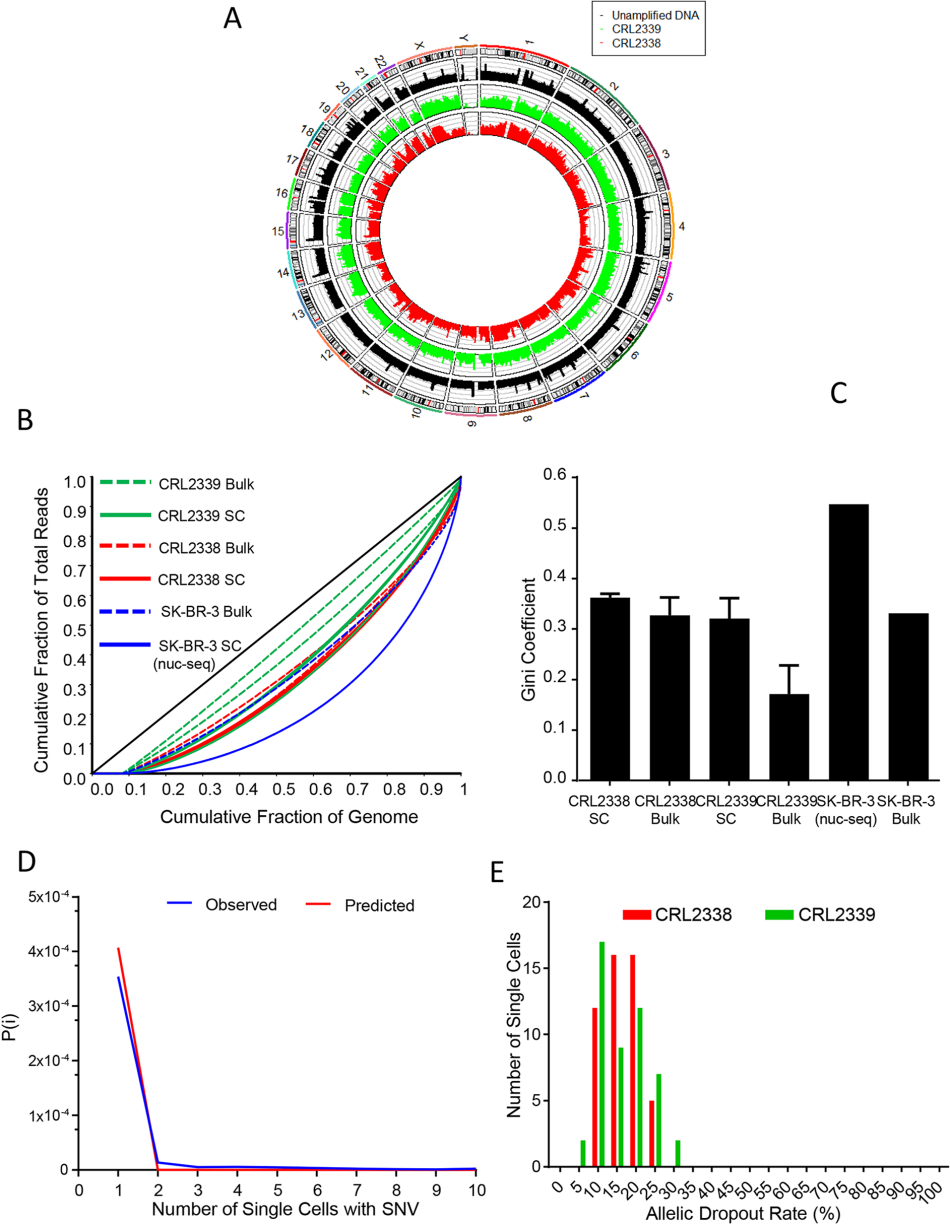
Single-cell WGS and WES of normal and cancer cells derived from the same individual. **(A)** Circos plot of normalized read counts (reads/100kb normalized to the total number of reads per sample) for one CRL2338/HCC1954 breast cancer cell, one CRL2339/HCC1954BL normal B-lymphoblast and a non-amplified bulk genomic DNA control. (**B)** Read distribution uniformity represented as Lorenz curves for individual CRL2338/HCC1954 cells (solid red, n = 3), CRL2339/HCC1954BL cells (solid green, n = 3), CRL2338/HCC1954 bulk genomic DNA (dotted red, n = 2), CRL2339/HCC1954BL bulk genomic DNA (dotted green, n = 2), a SK-BR-3 single cell (SK1, solid blue), and SK-BR-3 bulk genomic DNA (SKP, dotted blue) The ideal black diagonal line represents perfect uniformity. Samples labeled “TCGA” bulk genomic DNA controls for CRL2338/HCC1954 and CRL2339/HCC1954BL were sequenced independently by TCGA. (**C)** Gini coefficients (*G*) derived from the Lorenz curves to quantify the dispersion of reads throughout the genome for CRL2338/HCC1954 single cells (n = 3), CRL2339 single cells (n = 3), CRL2338/HCC1954 bulk genomic DNA (n = 2), CRL2339/HCC1954BL bulk genomic DNA (n = 2), SK-BR-3 single-cell (SK1, n = 1), and SK-BR-3 bulk genomic DNA control (SK-P, n = 1). *G* = 0 is perfect uniformity, *G* = 1 is complete non-uniformity. (**D)** Shown is the distribution of probabilities, *P(i)*, of a variant occurring in 1 or more cells, assuming a SNV FDR of 4.11x10^-6^. Given this SNV FDR, as the same variant is observed in multiple cells the probability of that variant being false decreases dramatically as it is found in more cells. € Allelic dropout rates observed in WES data for CRL2338/HCC1954 single cells (n = 50) and CRL2339/HCC1954BL single cells (n = 49). Loss of an allele in single-cell samples was quantified at 7,414 high-confidence heterozygous sites identified in bulk genomic DNA controls.

Intratumor genetic heterogeneity is a core feature of many types of cancers, yet is poorly understood at the single-cell level. In order to assess single-cell WGA within the context of genetically heterogeneous cancer cells, we applied our workflow to normal and breast cancer cell lines derived from the same individual. We used an established cell line from an ER-/PR-/HER2+ primary ductal carcinoma (CRL2338/HCC1954) as well as immortalized normal B-lymphoblasts isolated from the same patient as a control (CRL2339/HCC1954BL). Cell capture rates, WGA concordance with the presence of a live cell, WGA amplification yields, and WGA amplification gains were consistent with those observed with karyotypically normal GM12752 cells (S1 Table). Three single cells plus one genomic DNA control from each line were carried through WGS to an average depth of 23X. Across all six cells tested, breadth of genomic coverage (≥1X) reached an average of 90.24±1.29%, compared to 91.27% coverage in non-amplified bulk genomic DNA controls (Fig 2A). Lorenz plots also demonstrated improved amplification uniformity across the genome compared to nuc-seq (Fig 2B and 2C, CRL2338 G = 0.36±0.008, CRL2339 G = 0.32±0.04). Consistent with single-cell WGS on GM12752 cells, these results indicate that our implementation of WGA captures the vast majority of the genome accessible by a conventional WGS experiment in cancerous cells, with improved uniformity compared to current methods.

We next assessed the fidelity of WGA implemented in the C1 system, a critical factor influencing the ability to accurately identify SNVs. To do so, we examined the coding portion of a larger population of cells using whole exome enrichment and sequencing of libraries generated from 50 cells of each type. The single-cell exome-enriched DNA was sequenced to average coverage depth of ~27X per cell. Efficient exome enrichment was verified by comparing single-cell WES to sequencing results using exome enrichments from bulk genomic DNA controls (n = 2 per cell line). Read mapping rates across all single cells tested were consistently high (99.3%±0.15%, mean±SD) and were equivalent to those observed in bulk genomic DNA libraries (S2A Fig). Further, fold enrichment of on-target reads was similar between single-cells and bulk genomic DNA controls, indicating exome capture was efficient (S2B Fig). Exome coverage reached 88.1%±9.3% with ≥1X depth and 48.6%±9.5% with ≥10X depth, which differed by only 6.1% and 18.2%, respectively, from bulk genomic DNA (S2C Fig). Comparison of genotypes that were also called in the WGS data demonstrated 94.3%±2.5% (mean ±SD) SNV concordance with WES, confirming the accuracy of genotypes in the population of cells assayed by WES (S2C Fig).

SNV false discovery rate (FDR), evaluated at 5,487,871 high-confidence homozygous reference sites (see materials and methods), was 4.11x10^-6^ (S3 Table), a rate consistent with previously reported values for MDA-based WGA [4] and approximately an order of magnitude less than that observed in PCR-based WGA methods [22]. WGA amplification bias can lead to amplification of one allele at heterozygous sites, leading to allelic dropout (ADO). To quantify ADO rates we identified 7,414 high-confidence heterozygous sites (see materials and methods) in the bulk genomic DNA controls and determined the frequency with which one allele or the other was lost in each of the single cells assayed. Overall, ADO was 13.75%±0.53% (Fig 2E, mean±SD, see materials and methods). These data indicate that technical artifacts introduced by WGA are minimal using our approach, and compare favorably with previously reported single-cell WGA methods.

**Fig 3:**
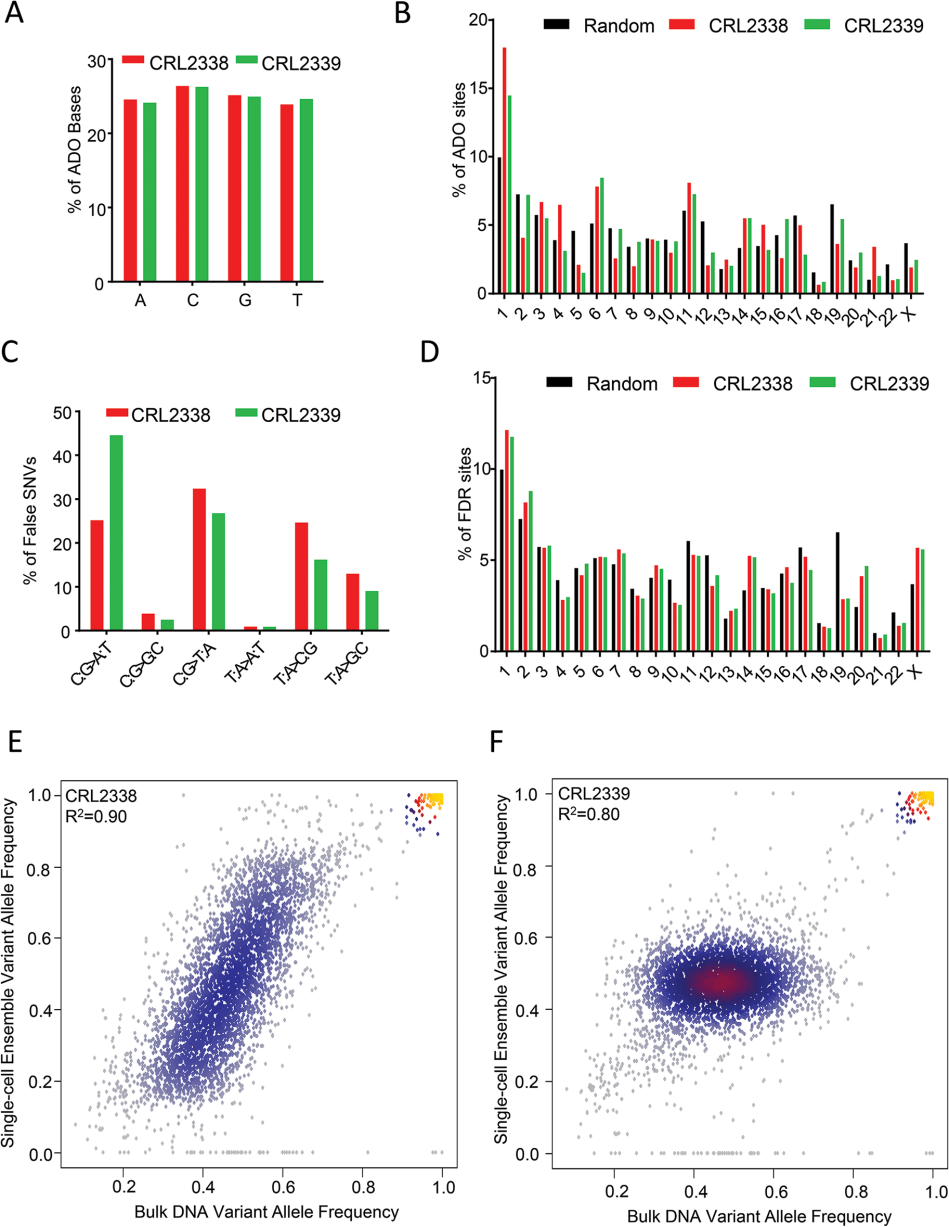
Characterization of C1 single-cell WGA artifacts. **(A)** The spectrum of base changes at ADO sites. Plotted is the mean percentage of ADO sites accounted for by each of the four bases across all CRL2338/HCC1954 (red, n = 50) and CRL2339/HCC1954BL (green, n = 49) cells. (**B)** The genomic distribution of ADO sites. Shown is the mean percentage of ADO sites on each chromosome compared to the expected distribution, assuming equal distribution of ADO sites across the genome (random, black) for CRL2338/HCC1954 (red, n = 50) and CRL2339/HCC1954BL (green, n = 49) cells. The random distribution is generated by equally distributing ADO sites across chromosomes based on the size of the targeted regions on each chromosome. (**C)** The spectrum of bases changes at false SNVs. Shown is the mean percentage of each possible base pair change at false SNVs identified in CRL2338/HCC1954 (red, n = 50) and CRL2339/HCC1954BL (green, n = 49). (**D)** The genomic distribution of false SNVs. Shown is the mean percentage of false SNVs on each chromosome compared to the expected distribution, assuming equal distribution of false SNVs across the genome (random, black) for CRL2338/HCC1954 (red, n = 50) and CRL2339/HCC1954BL (green, n = 49) cells. The random distribution is generated by equally distributing false SNVs across chromosomes based on the size of the targeted regions on each chromosome. (**E)** Comparison of the variant allele frequencies in bulk genomic to DNA to single-cell ensemble variant allele frequencies in CRL2338/HCC1954. Note that CRL2338/HCC1954 cells are near tetraploid by karyotype, which is expected to lead to VAFs ranging from 0.25–0.74 for heterozygous loci. (**F)** Comparison of the variant allele frequencies in bulk genomic to DNA to single-cell ensemble variant allele frequencies in normal diploid CRL2339/HCC1954BL cells.

One advantage of single-cell genetic analysis is the ability to leverage genotype information across the population of cells tested. Observing a variant in multiple cells reduces the chance of that variant being false if technical error is random. In order to characterize the technical artifacts potentially introduced during the amplification of DNA from individual cells, we further examined alleles that dropped out as well as falsely discovered variants. The spectrum of base changes found at ADO sites was equivalent for each of the four bases, suggesting it is in fact random (Fig 3A). Examination of the distribution of variants across the genome demonstrated that the ADO was also largely random, with the exception of a few chromosomes (chr1, chr6, chr14, chr22) (Fig 3B). This observation is consistent with those previously made where ADO rates were found to increase at localized regions of the genome that corresponded to sites proximal to telomeres and centromeres as a result of WGA biases [4]. Variant false discovery displayed a preference for C:G>T:A and C:G>A:T base changes (Fig 3C), a spectrum similar to that previously reported in multiple cancer types [4, 14]. False SNVs, however, were largely spread at random through the genome (Fig 3D). These findings indicate that although false SNVs are rare and spread randomly throughout the genome, there is some preference for base changes during the process of WGA, library preparation, and/or sequencing that are important to take into consideration.

Overall, SNV FDR and ADO rates were comparably low during WGA using the C1 system, indicating that genotype information at the single-cell level was representative of that observed when genotyping from bulk genomic DNA. To confirm this, we also quantified single-cell ensemble variant allele frequencies (VAFs) for each cell type and determined the overall correlation with VAFs observed in the bulk genomic DNA controls (Fig 3E and 3F). Single-cell ensemble VAFs were highly correlated with those found in bulk genomic DNA in both normal CRL2339/HCC1954BL (R^2^ = 0.80) and cancer CRL2338/HCC1954 cells (R^2^ = 0.90) (Fig 3E and 3F). Correlations were similarly high for allele frequencies at all genotyped sites (R^2^ = 0.87 in CRL2338/HCC1954, R^2^ = 0.86 in CRL2339/HCC1954BL) (S3A and S3B Fig). Interestingly, the VAF distributions for heterozygous sites in CRL2338/HCC1954 and CRL2339/HCC1954BL were clearly distinct. For normal CRL2339/HCC1954BL cells, the distribution clustered tightly around 0.5, but ranged between ~0.2–0.8 in CRL2338/HCC1954 cells. CRL2338/HCC1954 is a near tetraploid cell line [24], which is expected to lead to a VAF distribution at heterozygous sites that ranges from ~0.25–0.75. This is in contrast to the distribution expected at heterozygous sites in normal diploid cells, which should center at ~0.5, similar to the observed distribution in the normal CRL2339/HCC1954BL cells. The combination of high correlations between the single-cell ensemble allele frequencies and bulk genomic DNA, as well as the distinct allele frequency distributions in diploid and near tetraploid cells, reflects the accuracy of the WGA process across the population of single-cell tested, indicating accurate genotypes are accessible at the single-cell level.

**Fig 4:**
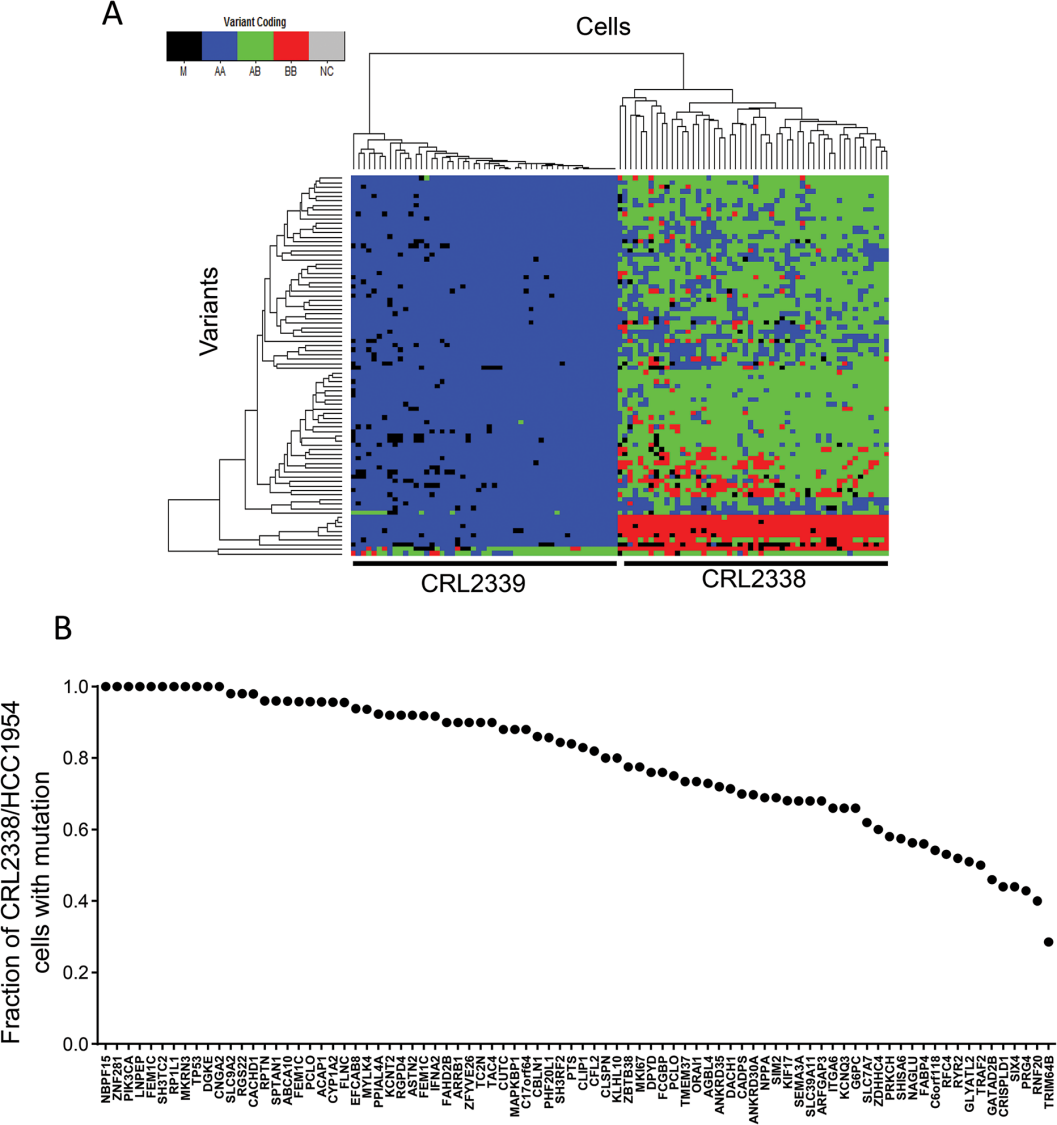
Single-cell genetic variation in CRL2338/HCC1954, ER-/PR-/HER2+ breast cancer cells. **(A)** Heatmap representations of the validated set of 84 mutations in 81 genes identified in CRL2338/HCC1954 cells. Shown on the left are genotypes for the CRL2339/HCC1954BL cells (n = 49) and on the right genotypes for the 50 CRL2338/HCC1954 cells (n = 50). Genotype information is encoded as: black–no genotype call (M); blue–homozygous reference (HOM REF); green–heterozygous (HET); and red–homozygous variant (HOM ALT). Both cells and variants are clustered hierarchically based on Hamming distance. (**B)** The frequency of variants in the CRL2338/HCC1954 single-cell population (n = 50).

We next determined the probability of observing a variant in one or more cells by applying the SNV FDR to a binomial test (cumulative distribution function), as previously reported[4]. Using this approach, we find that the probability, *P(i)*, of a variant being false drops substantially when observed in ≥ 3 cells (S3 Table). Applying a threshold of ≥ 3 cells (*P(i)* = 1.09x10^-11^) identified 323 somatic mutations that occurred more frequently within CRL2338/HCC1954 breast cancer cells compared to the normal controls (S4A Fig and materials and methods). When considering the region targeted by WES, this threshold translates to less than one false variant per single cell (2.59x10^-4^) (S3 Table). Further, hierarchical clustering of individual cells based on the mutation profile clearly distinguished the population of cancer cells from the matched normal cells (S4 Fig). Genetic analysis with single-cell resolution provides the unique ability to distinguish co-segregating somatic mutations amongst cells in order to resolve underlying heterogeneity. In order to determine how somatically acquired mutations were distributed amongst individual cells we first focused on validated mutations that were previously identified in bulk genomic DNA isolated from CRL2338/HCC1954 cells [25]. This comparison yielded 84 non-synonymous mutations within 81 genes (S4 Table). Additional validation using targeted resequencing of a known *TP53* mutation in each of the 100 cells tested confirmed the mutation in each CRL2338/HCC1954 cell, while none of the normal CRL2339/HCC1954BL cells were found to harbor the mutation, further reflecting the accuracy of the set of mutations identified. Hierarchical clustering of samples did not identify a clear subclonal architecture within the population. However, using this set of mutations, we found that no two cells were identical based on their mutation profile, highlighting the presence of underlying heterogeneity within the tumor cells (Fig 4A). This heterogeneity was not simply due to technical artifacts, since the normal B-lymphoblast controls did not show this behavior: all normal B-Lymphoblasts had the same genotype (Fig 4A) for 79 of 84 mutations present in tumor cells.

Heterogeneity within the tumor cell population was highlighted by variation in the fraction of cells harboring known cancer-related mutations (Fig 4B). For instance, 100% of the tumor cells assayed harbored known mutations in *TP53* and *PIK3CA*, two key driver genes in diverse cancer types. Yet, only 52% of these cells harbored mutations in *RYR2* (Fig 4B). Also of note was that, among the 323 mutations identified, several that were not previously found in CRL2338/HCC1954 cells but that are known to be mutated in various other types of cancers [15] were identified using only genotype data from single cells. These genes included *ZFHX3* (100% of cells), *SACS* (62% of cells), *NBPF1* (60% of cells), *FLG* (34% of cells), and *ZNF471* (31% of cells). These results support the notion that novel somatic mutations can in fact arise in cancer related genes over time and potentially implicate mutations in these genes as contributing to the intratumor genetic heterogeneity of ER-/PR-/HER2+ breast cancer.

**Fig 5:**
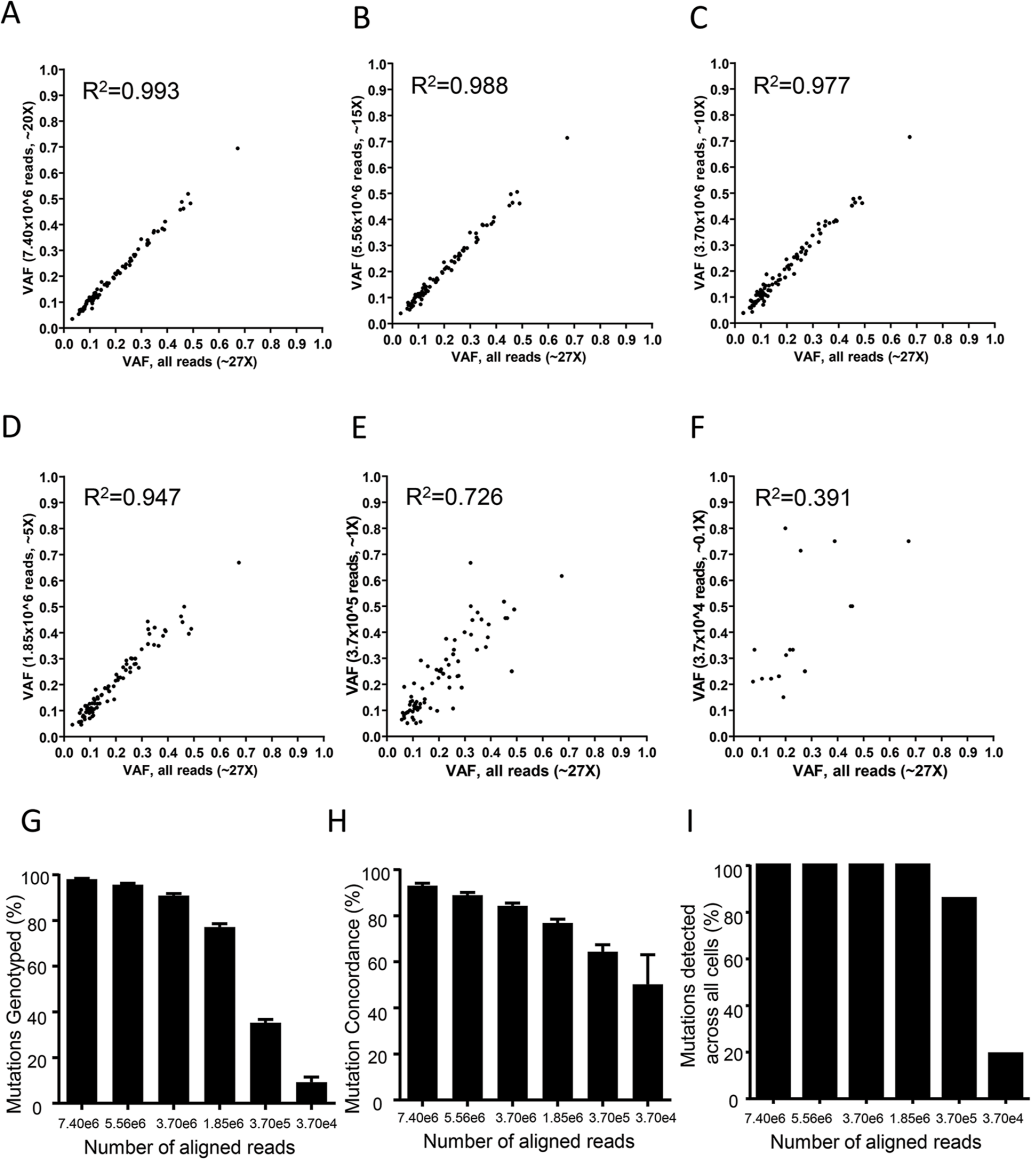
Low-depth WES mutation profiles are sufficient to detect high-confidence mutations. **(A-F)** Single-cell ensemble VAF correlations between the full WES dataset (~27X depth) and WES datasets with reads down-sampled to 7.40x10^6^ (**A**, ~20X depth), 5.56x10^6^ (**B**, ~15X depth), 3.70x10^6^ (**C**, ~10X depth), 1.85x10^6^ (**D**, ~5X depth), 3.7x10^5^ (**E**, ~1X depth), and 3.7x10^4^ (**F**, ~0.1X depth). R^2^ values are indicated in the top left corner. (**G)** The percentage of mutations at which genotypes were called in the full WES dataset (~27X depth) and WES datasets with reads down-sampled to 7.40x10^6^ (~20X depth), 5.56x10^6^ (~15X depth), 3.70x10^6^ (~10X depth), 1.85x10^6^ (~5X depth), 3.7x10^5^ (~1X depth), and 3.7x10^4^ (~0.1X depth). (**H)** The concordance in mutation calls between the full WES dataset (~27X depth) and WES datasets with reads down-sampled to 7.40x10^6^ (~20X depth), 5.56x10^6^ (~15X depth), 3.70x10^6^ (~10X depth), 1.85x10^6^ (~5X depth), 3.7x10^5^ (~1X depth), and 3.7x10^4^ (~0.1X depth). (**I)** The percentage of mutations identified in at least one cell using the full WES dataset as well as WES datasets with reads down-sampled to 7.40x10^6^ (~20X depth), 5.56x10^6^ (~15X depth), 3.70x10^6^ (~10X depth), 1.85x10^6^ (~5X depth), 3.7x10^5^ (~1X depth), and 3.7x10^4^ (~0.1X depth). For all data the values were determined for the set of 84 validated mutations identified in CRL2338/HCC1954 cells.

**Fig 6:**
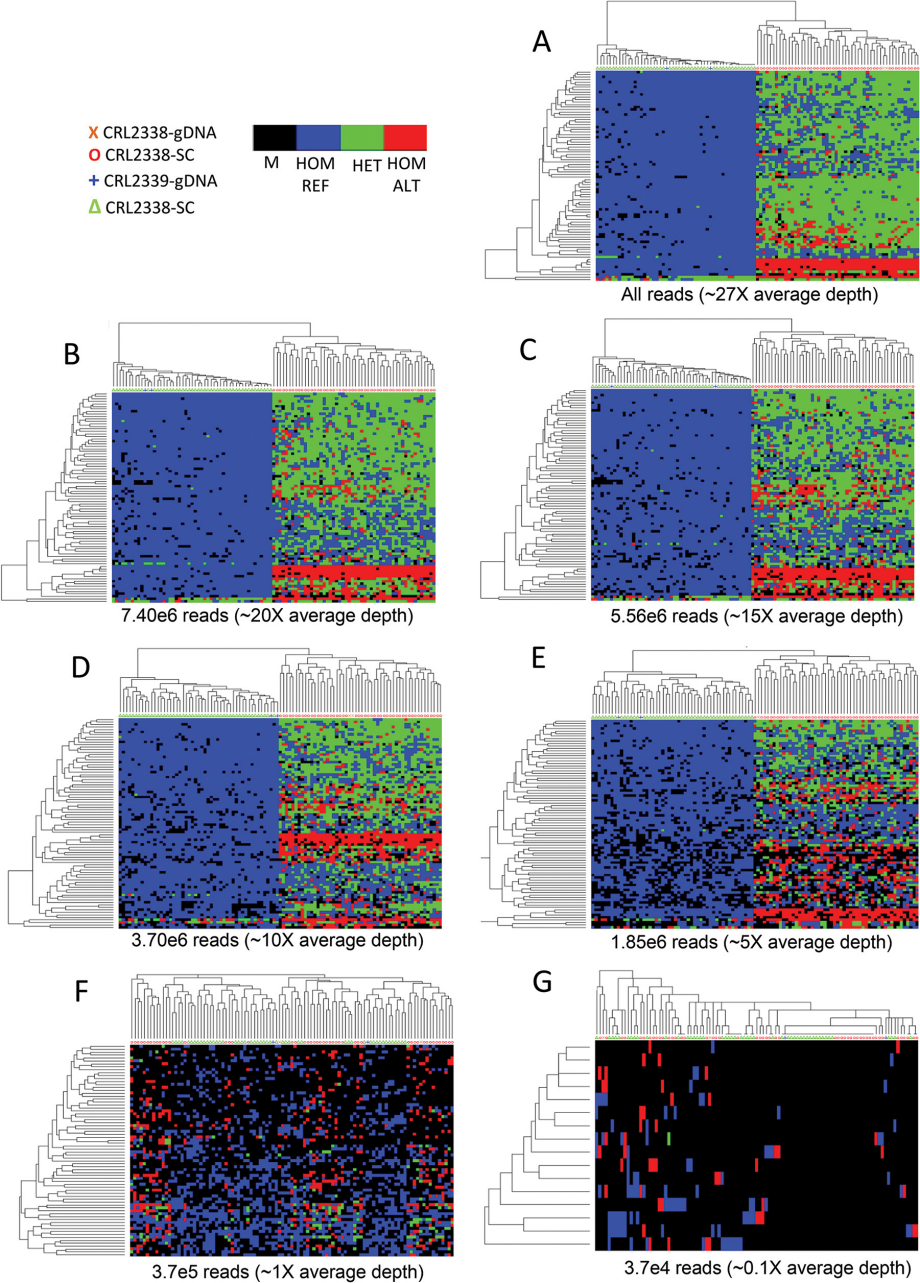
Low-depth WES mutation profiles are sufficient to distinguish tumor from normal cells. (**A-G)** Heatmap representations of the set of 84 validated mutations identified in the full CRL2338/HCC1954 WES dataset (**A,** ~27X depth) as well as WES datasets with reads down-sampled to 7.40x10^6^ (**B**, ~20X depth), 5.56x10^6^ (**C**, ~15X depth), 3.70x10^6^ (**D**, ~10X depth), 1.85x10^6^ (**E**, ~5X depth), 3.7x10^5^ (**F**, ~1X depth), and 3.7x10^4^ (**G**, ~0.1X depth). Genotype information is encoded as: black–no genotype call (M); blue–homozygous reference (HOM REF); green–heterozygous (HET); and red–homozygous variant (HOM ALT).

We examined the effect of sequencing depth on mutation calling and cell classification using our WES data from our population of single cells. To do so, we down-sampled reads and then quantified VAF correlations, detection sensitivity, and genotype concordance at mutations identified amongst the 50 CRL2338/HCC1954 cells. Single-cell ensemble VAFs in the down-sampled data remained highly correlated with down to ~10X average exome coverage (Fig 5A–5F, 3.7x10^6^ reads, R^2^ = 0.977), indicating accurate genotype information was obtained with this level of sequencing. In line with this, we observed 91.0% detection sensitivity (Fig 5G) while maintaining 84.3% concordance in mutation calls (Fig 5H) at ~10X average coverage (3.7x10^6^ reads). Similar sensitivity, genotype concordance, and VAF correlation was observed for the full set of 323 mutations identified (S5A–S5H Fig). Subsequent hierarchical clustering based on Hamming distance demonstrated that CRL2338/HCC1954 tumor cells could be classified with 100% accuracy down to 5X average sequencing depth using both sets of mutations (Fig 6A–6G and S6A–S6G Fig). Also of note is that when considering mutation calls across the entire population of single cells tested, 100% of the 84 validated mutations were detected in at least one cell with only 5X average coverage of the exome, which indicates that low-depth sequencing is still capable of identifying mutations present in the populations of cells tested with high sensitivity (Fig 5I). Together, these data demonstrate the benefit of utilizing low-depth population-based sequencing for mutational profiling in single cells to both identify key mutations and effectively classify cancer cells.

## Discussion

In this study we report the development of an automated hands-free workflow for the capture, lysis, and amplification of genomic DNA from single cells using nanoliter-scale microfluidics. We demonstrate improvements in cell capture efficiency and sample cross-contamination over previously reported “cell seeding”-based methods for single-cell isolation and WGA [16, 26]. Although in this study we have focused on WGS and WES of DNA amplified from single-live cells (as validated by microscopy and LIVE/DEAD cell staining), we have observed particular failure modes for cell capture sites that either lack a cell altogether or a single-dead cell. Specifically, these include low levels of amplified DNA that when sequenced were found to correspond to mitochondrial DNA as well as repetitive regions of the genome. Additionally, we find that careful annotation of captured cells by microscopy is an important step to prevent analysis of DNA derived from capture sites in which multiple cells were captured.

Effective genetic analysis with single-cell resolution requires that the genome of each cell be amplified with breadth, uniformity, and high fidelity. In both karyotypically normal and affected tumor cell lines, ~90%-95% of the genome accessible through conventional bulk genomic DNA WGS is covered by single-cell WGS. With the same number of aligned reads, this breadth of coverage was 8.8% greater than observed in nuc-seq (relative to matched bulk genomic DNA controls). Further, read distribution uniformity was also improved relative to nuc-seq on SK-BR-3 cells, which are near triploid (corresponding to ~6N genomic content in G2/M cells) Although we observed similar performance in normal diploid B-lymphoblasts and near tetratploid ER-/PR-/HER2+ CRL2338 cells, moving forward it may be interesting to assess genomic coverage breadth and amplification uniformity of G2/M cells using the C1 system. The differences in coverage breadth and uniformity are likely attributable to a combination of performing the MDA reaction at the nanoliter scale, increasing the Φ29 isothermal amplification temperature to 38°C (typically 30°C), and by controlling MDA reaction gains (2.9–4.6x10^4^ on average across cell types), consistent with previous observations using WGA of bacterial DNA [22].

A known strength of single-cell WGA using Φ29-based MDA is the high fidelity of the enzyme. We systematically identified technical artifacts introduced by Φ29 during amplification by quantifying SNV FDRs and ADO rates, finding that each of these is in fact relatively limited Consistent with the relatively low SNV FDRs and ADO rates, we find that single-cell ensemble allele frequencies are highly correlated with those found in bulk genomic DNA. This subsequently allowed the probability of a variant being false to be determined as a function of the number of cells that variant was observed in. Importantly, the combination of these observations enabled the use of genotypes exclusively from single cells without the need to necessarily anchor analyses on sequencing of bulk genomic DNA from the same source.

By applying this method to matched tumor and normal cells derived from an ER-/PR-/HER2+ cell line, and a matched normal control cell line we were able to identify 323 somatic mutations that were present in ≥ 3 cells and clearly distinguished all tumor cells from all normal cells using hierarchical clustering. Within this set, 84 mutations in 81 genes were previously identified in the same cell line using bulk genomic DNA, including mutations in multiple key cancer driver genes such as *TP53* and *PIK3CA*. We focused our analysis on this set of 84 validated mutations to establish how known somatically acquired mutations can be differentially distributed amongst individual cells. Using this approach, it was clear that extensive heterogeneity existed across the population of single cells assayed, as no two cells had identical mutation profiles. This heterogeneity was not simply due to technical artifacts, since all normal B-lymphoblasts had the same genotype at 79/84 mutations identified in tumor cells. Yet no clear subclonal genetic architecture could be discerned. The lack of subclonal heterogeneity is consistent with previous studies on bulk genomic DNA using the same cell line. However, it is possible that further experiments on larger numbers of cells could reveal rare subclones. In total, 11 mutations in 11 different genes were found in 100% of the cancerous cells. However, even amongst this subset of validated mutations, there were an additional 73 mutations in 70 genes that were present in only a subset of cells (ranging from 28.6% to 98% of cells). This suggests a substantial amount of single-cell genetic heterogeneity in mutations previously identified from bulk genomic DNA for which segregation patterns could not otherwise be resolved at the level of a single cell. While bulk genomic DNA has been used to infer clonal architectures [1, 10, 27, 28], such an approach is limited in its ability to truly resolve somatic mosaicism and identify co-segregation of mutations within the same cell.

Population-scale genetic studies using high-throughput sequencing of bulk genomic DNA have established that as progressively larger numbers of samples are sequenced, increasingly accurate genotype information can be derived using shallow sequencing depths, down to 4-10X average coverage [29, 30]. Single-cell genetic analysis is in essence population-based genetic analysis. Thus, we find that using our method, sequencing depth could be limited to ~10X average exome coverage while maintaining high mutation-detection sensitivity and specificity. Further, cell classification based on the mutations profiles obtained with low-depth sequencing down to 5X average coverage was 100% accurate, clearly distinguishing all tumor cells from the matched normal controls. Although genotyping sensitivity does decrease slightly with lower sequencing depths, we find that when looking across the population of cells tested, 100% of mutations identified at ~27X average coverage were identified in at least one cell with down to ~5X average coverage. These results point toward the benefits of study designs in which sequencing efforts are distributed across larger numbers of cells rather than requiring high sequencing depths in fewer numbers of individual cells.

Together, our data demonstrate an efficient automated microfluidic platform for single-cell WGA that enables distinction of mutation segregation patterns in populations of genetically heterogeneous cells.

## Supporting Information

S1 Fig WGA amplicon size distribution. (**A**) Plotted is the size distribution of WGA amplicons from a representative subset of five GM12752 single cells. (**B**) Comparison of WGA amplicons derived from a single live cell, verified by LIVE/DEAD cell staining, and WGA amplicons derived from an empty C1 IFC capture site.
(TIF)

S2 Fig Summary of single-cell WES on CRL2338 and CRL2339 cells. **(A)** Total read yields and read alignment rates for CRL2338 cells (n = 50) and CRL2339 cells (n = 49). The percentage of aligned reads per cell is plotted on the left axis (blue) and total read yield per cell is plotted on the right axis (black). Bulk genomic DNA controls from CRL2339 (n = 2, green) and CRL2338 (n = 2, red) are highlighted on the far left for comparison. (**B**) Fold enrichment of on-target reads over off-target reads for WES from each individual cell. Bulk genomic DNA controls from CRL2339 (n = 2, green) and CRL2338 (n = 2, red) are highlighted on the far left for comparison. (**C**) SNV concordance between variants identified in both WGS and WES experiments.
(TIF)

S3 Fig Comparison of the allele frequencies in bulk genomic DNA to single-cell ensemble allele frequencies. (**A**) Allele frequencies at all genotyped sites in bulk genomic DNA controls compared to the single-cell ensemble allele frequencies calculated from CRL2338 cells (n = 50). (**B**) Allele frequencies at all genotyped sites in bulk genomic DNA controls compared to the single-cell ensemble allele frequencies calculated from CRL2339 cells (n = 49).
(TIF)

S4 Fig Heat map representations of the 323 mutations identified from CRL2338 single-cell WES. Shown on the left are genotypes for the CRL2339 cells (n = 49) and on the right genotypes for the CRL2338 cells (n = 50). Genotype information is encoded as: black, no genotype call (M); blue, homozygous reference (HOM REF); green, heterozygous (HET); and red, homozygous variant (HOM ALT). Both cells and variants are clustered hierarchically based on Hamming distance.
(TIF)

S5 Fig Low-depth WES mutation profiles are sufficient to detect high-confidence mutations. (**A-F**) Single-cell ensemble VAF correlations between the full WES dataset (~27X depth) and WES datasets with reads down-sampled to 7.40x10^6^ (**A**, ~20X depth), 5.56x10^6^ (**B**, ~15X depth), 3.70x10^6^ (**C**, ~10X depth), 1.85x10^6^ (**D**, ~5X depth), 3.7x10^5^ (**E**, ~1X depth), and 3.7x10^4^ (**F**, ~0.1X depth). R^2^ values are indicated in the top left corner. (**G**) The percentage of mutations at which genotypes were called in the full WES dataset (~27X depth) and WES datasets with reads down-sampled to 7.40x10^6^ (~20X depth), 5.56x10^6^ (~15X depth), 3.70x10^6^ (~10X depth), 1.85x10^6^ (~5X depth), 3.7x10^5^ (~1X depth), and 3.7x10^4^ (~0.1X depth). (**H)** The concordance in mutation calls between the full WES dataset (~27X depth) and WES datasets with reads down-sampled to 7.40x10^6^ (~20X depth), 5.56x10^6^ (~15X depth), 3.70x10^6^ (~10X depth), 1.85x10^6^ (~5X depth), 3.7x10^5^ (~1X depth), and 3.7x10^4^ (~0.1X depth). (**I)** The percentage of mutations identified in at least one cell using the full WES dataset as well as WES datasets with reads down-sampled to 7.40x10^6^ (~20X depth), 5.56x10^6^ (~15X depth), 3.70x10^6^ (~10X depth), 1.85x10^6^ (~5X depth), 3.7x10^5^ (~1X depth), and 3.7x10^4^ (~0.1X depth). For all data the values were determined for the set of 323 mutations identified in CRL2338/HCC1954 cells.
(TIF)

S6 Fig Low-depth WES mutation profiles are sufficient to distinguish tumor from normal cells. (**A-G)** Heat map representations of the set of 323 mutations identified in the full CRL2338/HCC1954 WES dataset (**A,** ~27X depth) as well as WES datasets with reads down-sampled to 7.40x10^6^ (**B**, ~20X depth), 5.56x10^6^ (**C**, ~15X depth), 3.70x10^6^ (**D**, ~10X depth), 1.85x10^6^ (**E**, ~5X depth), 3.7x10^5^ (**F**, ~1X depth), and 3.7x10^4^ (**G**, ~0.1X depth). Genotype information is encoded as: black, no genotype call (M); blue, homozygous reference (HOM REF); green, heterozygous (HET); and red, homozygous variant (HOM ALT).
(TIF)

S1 Table Summary of C1 single cell capture and WGA. A summary of the number of C1 IFCs run, cell capture rates, and WGA yields.
(XLSX)

S2 Table Sample Accessions for Single-Cell Whole Genome and Whole Exome Sequencing. A summary of the sequencing datasets produced and analyzed, including the NCBI SRA accessions.
(XLSX)

S3 Table SNV FDR and Probabilities of Observing False Variants. A summary of the observed and predicted SNV FDRs and the associated probabilities of a given variant being false for this study.
(XLSX)

S4 Table Mutations identified from CRL2338/HCC1954 single-cells. A summary of the mutations identified in CRL2338/HCC1954 cells as well as the mutations previously identified using bulk genomic DNA from canSAR. Also included are mutations previously identified across cancer types (cancer5000).
(XLSX)
