# Supplementary figures and images for: Single-Cell Genetic Analysis Using Automated Microfluidics to Resolve Somatic Mosaicism
Source: PLoS One. 2015 Aug 24;10(8):e0135007. doi: 10.1371/journal.pone.0135007 (PMC4547741; doi:10.1371/journal.pone.0135007)

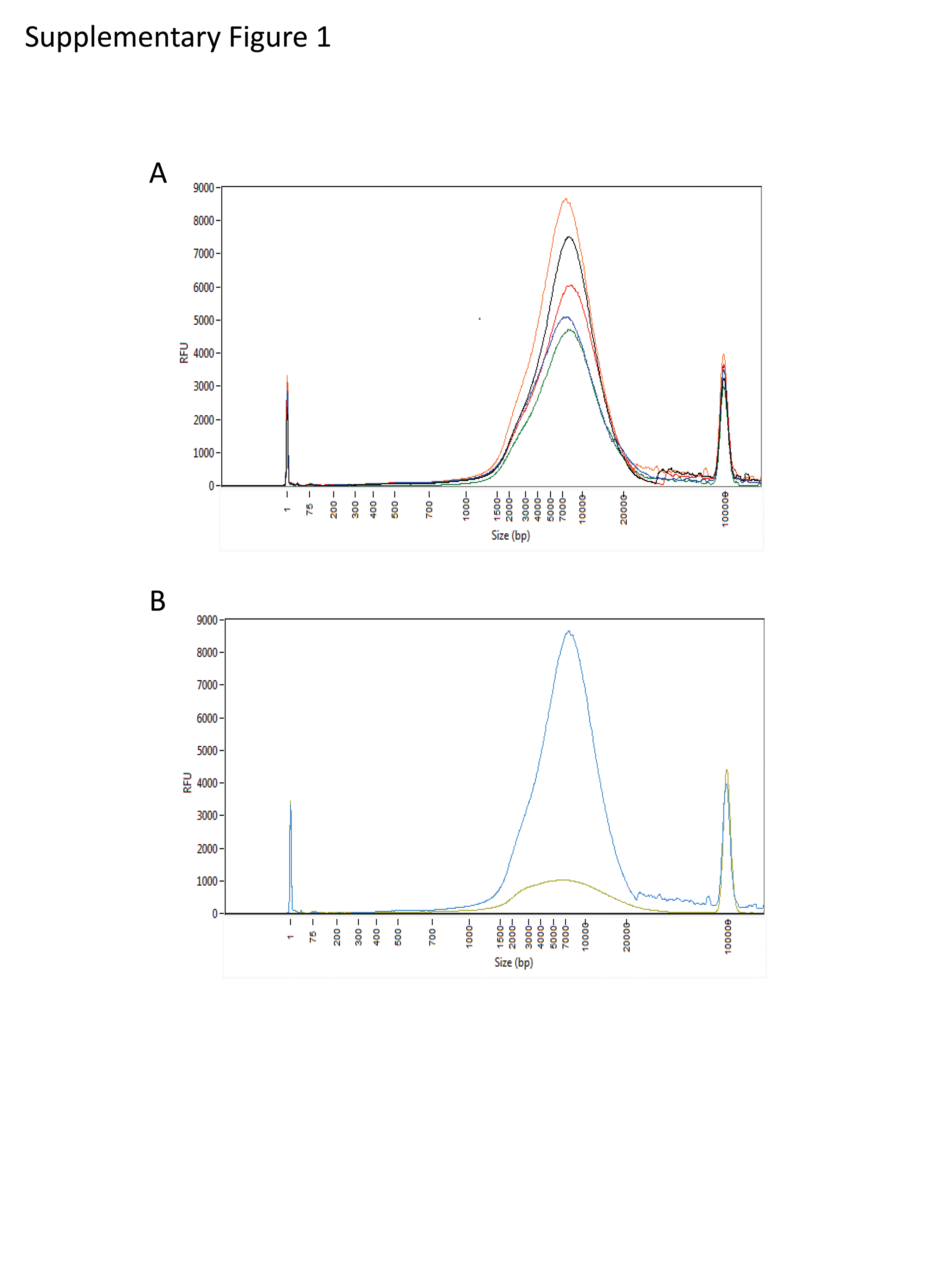

Supplement: S1 Fig — (A) Plotted is the size distribution of WGA amplicons from a representative subset of five GM12752 single cells. (B) Comparison of WGA amplicons derived from a single live cell, verified by LIVE/DEAD cell staining, and WGA amplicons derived from an empty C1 IFC capture site. (TIF) [file pone.0135007.s001.tif]

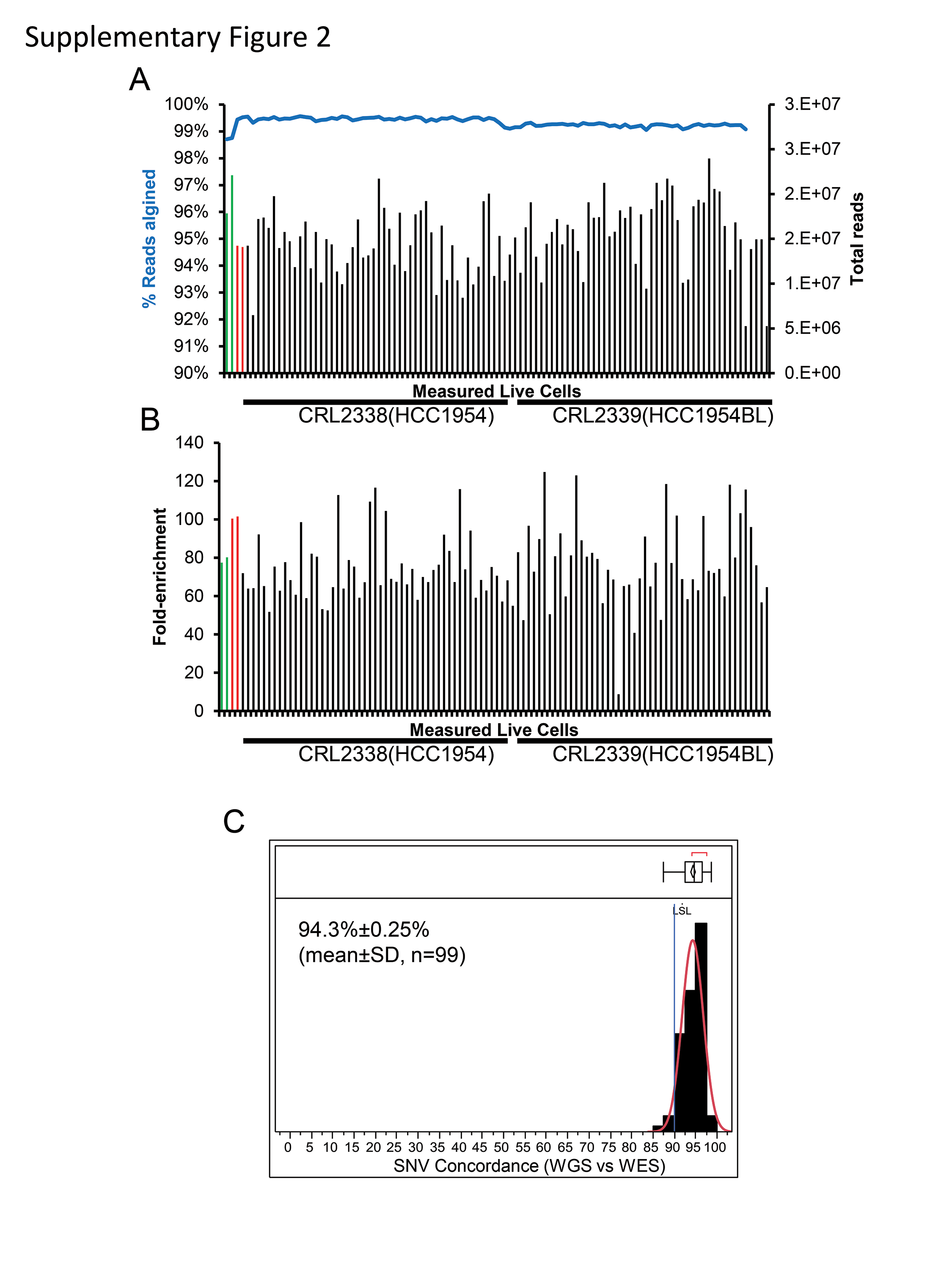

Supplement: S2 Fig — (A) Total read yields and read alignment rates for CRL2338 cells (n = 50) and CRL2339 cells (n = 49). The percentage of aligned reads per cell is plotted on the left axis (blue) and total read yield per cell is plotted on the right axis (black). Bulk genomic DNA controls from CRL2339 (n = 2, green) and CRL2338 (n = 2, red) are highlighted on the far left for comparison. (B) Fold enrichment of on-target reads over off-target reads for WES from each individual cell. Bulk genomic DNA controls from CRL2339 (n = 2, green) and CRL2338 (n = 2, red) are highlighted on the far left for comparison. (C) SNV concordance between variants identified in both WGS and WES experiments. (TIF) [file pone.0135007.s002.tif]

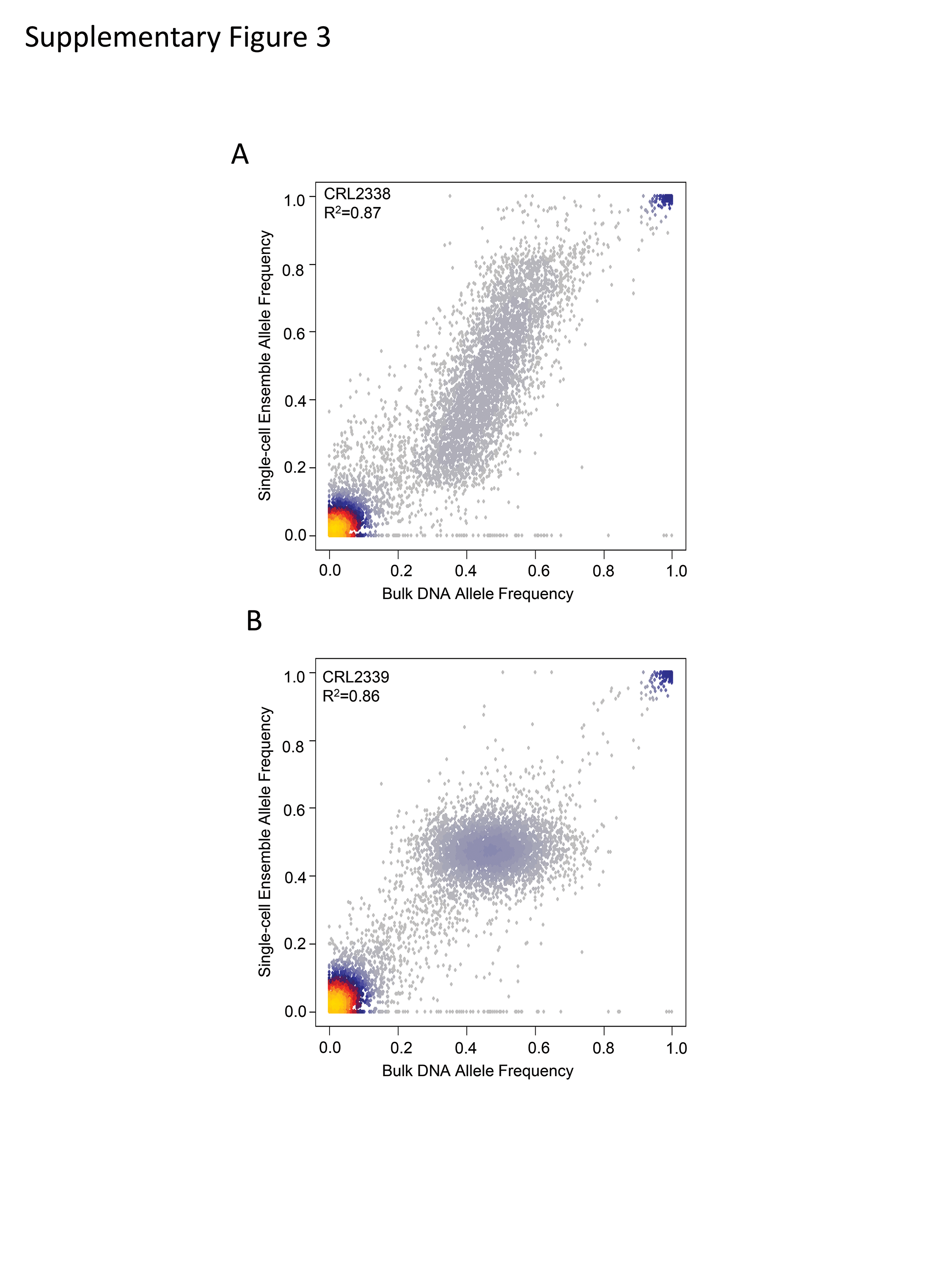

Supplement: S3 Fig — (A) Allele frequencies at all genotyped sites in bulk genomic DNA controls compared to the single-cell ensemble allele frequencies calculated from CRL2338 cells (n = 50). (B) Allele frequencies at all genotyped sites in bulk genomic DNA controls compared to the single-cell ensemble allele frequencies calculated from CRL2339 cells (n = 49). (TIF) [file pone.0135007.s003.tif]

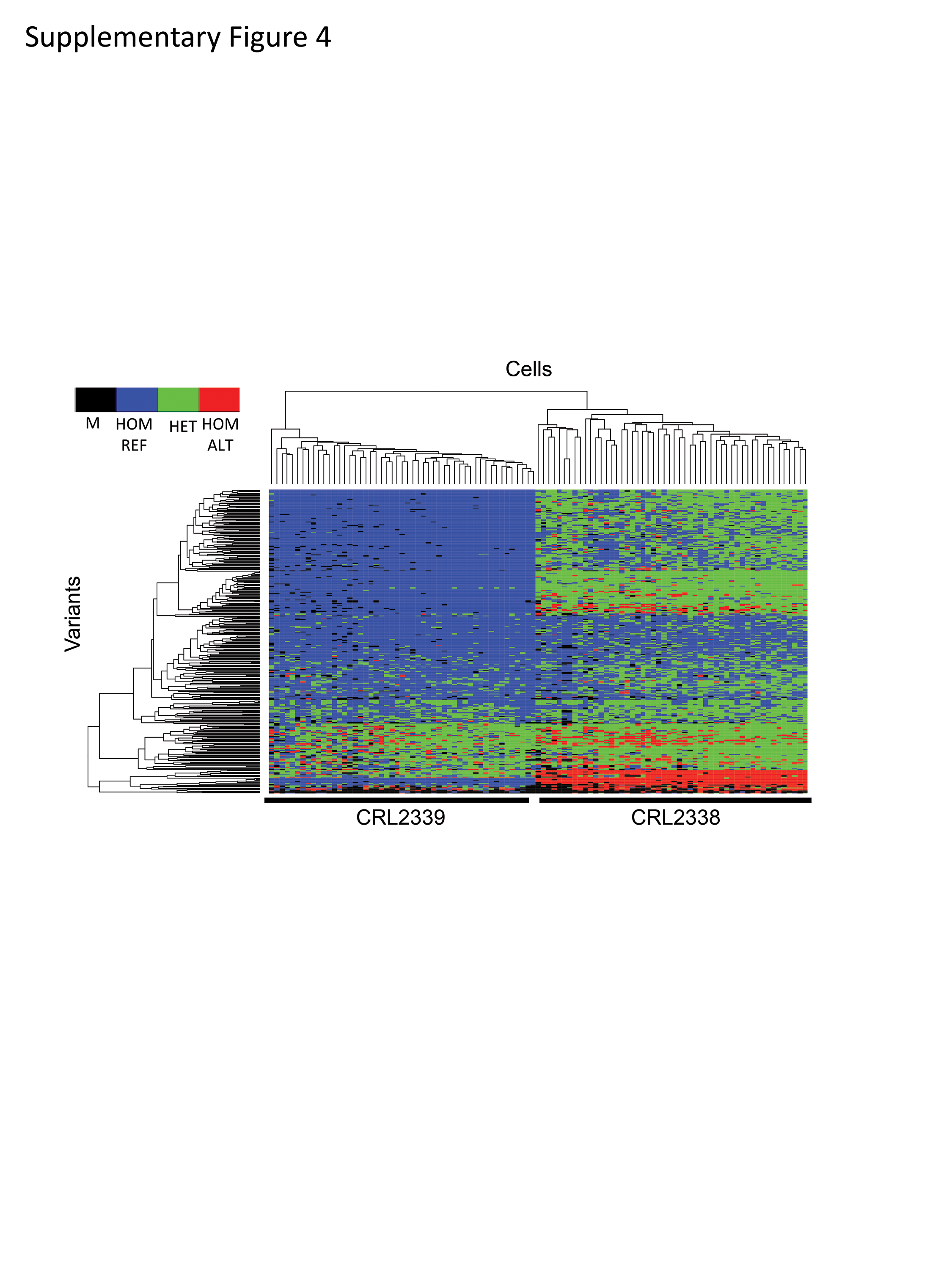

Supplement: S4 Fig — Shown on the left are genotypes for the CRL2339 cells (n = 49) and on the right genotypes for the CRL2338 cells (n = 50). Genotype information is encoded as: black, no genotype call (M); blue, homozygous reference (HOM REF); green, heterozygous (HET); and red, homozygous variant (HOM ALT). Both cells and variants are clustered hierarchically based on Hamming distance. (TIF) [file pone.0135007.s004.tif]

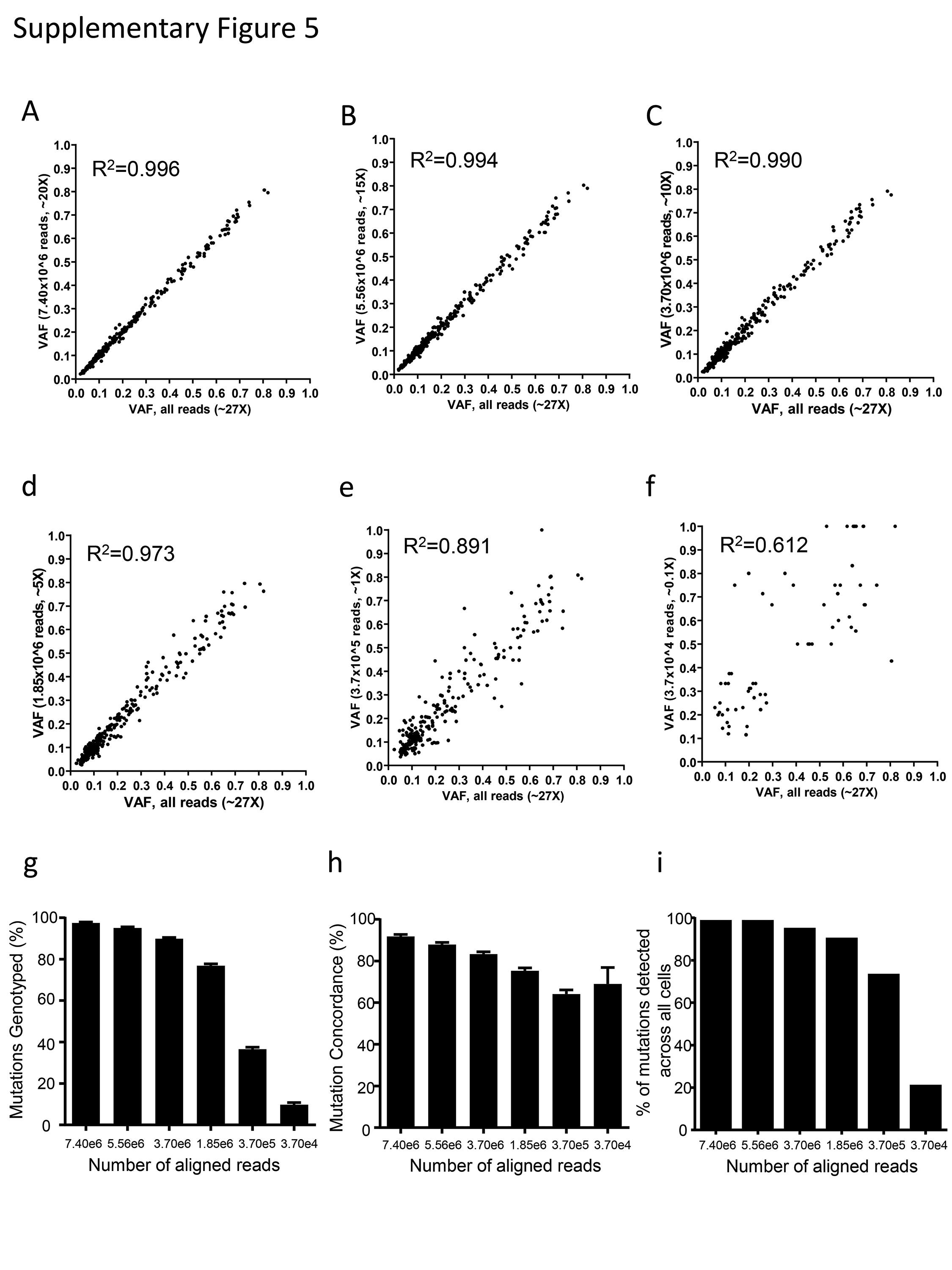

Supplement: S5 Fig — (A-F) Single-cell ensemble VAF correlations between the full WES dataset (~27X depth) and WES datasets with reads down-sampled to 7.40x10^6^ (A, ~20X depth), 5.56x10^6^ (B, ~15X depth), 3.70x10^6^ (C, ~10X depth), 1.85x10^6^ (D, ~5X depth), 3.7x10^5^ (E, ~1X depth), and 3.7x10^4^ (F, ~0.1X depth). R2 values are indicated in the top left corner. (G) The percentage of mutations at which genotypes were called in the full WES dataset (~27X depth) and WES datasets with reads down-sampled to 7.40x10^6^ (~20X depth), 5.56x10^6^ (~15X depth), 3.70x10^6^ (~10X depth), 1.85x10^6^ (~5X depth), 3.7x10^5^ (~1X depth), and 3.7x10^4^ (~0.1X depth). (H) The concordance in mutation calls between the full WES dataset (~27X depth) and WES datasets with reads down-sampled to 7.40x10^6^ (~20X depth), 5.56x10^6^ (~15X depth), 3.70x10^6^ (~10X depth), 1.85x10^6^ (~5X depth), 3.7x10^5^ (~1X depth), and 3.7x10^4^ (~0.1X depth). (I) The percentage of mutations identified in at least one cell using the full WES dataset as well as WES datasets with reads down-sampled to 7.40x10^6^ (~20X depth), 5.56x10^6^ (~15X depth), 3.70x10^6^ (~10X depth), 1.85x10^6^ (~5X depth), 3.7x10^5^ (~1X depth), and 3.7x10^4^ (~0.1X depth). For all data the values were determined for the set of 323 mutations identified in CRL2338/HCC1954 cells. (TIF) [file pone.0135007.s005.tif]

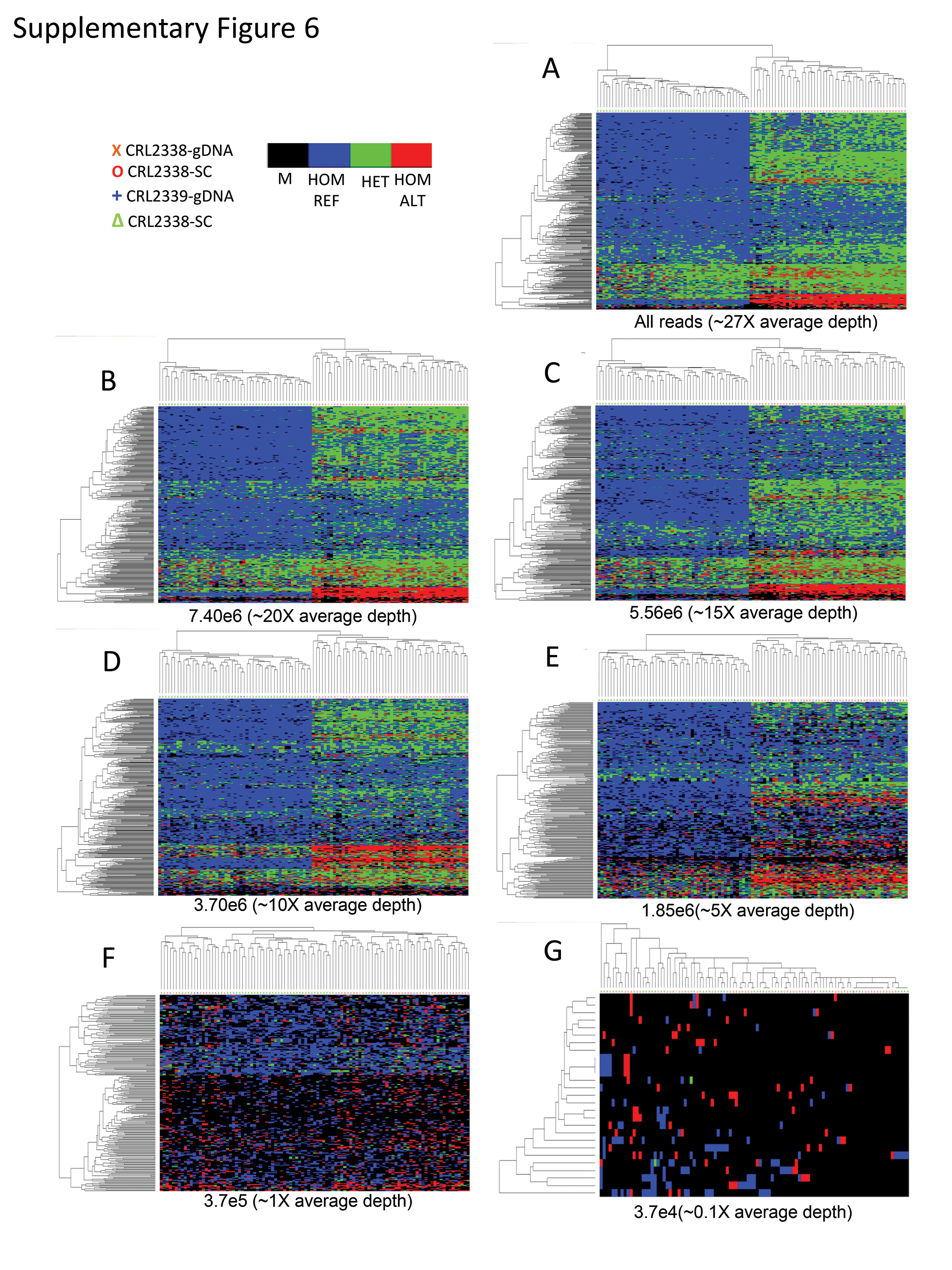

Supplement: S6 Fig — (A-G) Heat map representations of the set of 323 mutations identified in the full CRL2338/HCC1954 WES dataset (A, ~27X depth) as well as WES datasets with reads down-sampled to 7.40x10^6^ (B, ~20X depth), 5.56x10^6^ (C, ~15X depth), 3.70x10^6^ (D, ~10X depth), 1.85x10^6^ (E, ~5X depth), 3.7x10^5^ (F, ~1X depth), and 3.7x10^4^ (G, ~0.1X depth). Genotype information is encoded as: black, no genotype call (M); blue, homozygous reference (HOM REF); green, heterozygous (HET); and red, homozygous variant (HOM ALT). (TIF) [file pone.0135007.s006.tif]
